# Supplementary material for: Discontinuous transition to loop formation in optimal supply networks
Source: Nat Commun. 2020 Nov 16;11:5796. doi: 10.1038/s41467-020-19567-2 (PMC7670464; doi:10.1038/s41467-020-19567-2)
Supplement: Supplementary file 1 — Supplementary Information [file 41467_2020_19567_MOESM1_ESM.pdf]

Supplementary Information for
"Discontinuous transition to loop formation in optimal supply networks"

Franz Kaiser,[1,2,*] Henrik Ronellenfitsch,[3,4] and Dirk Witthaut[1,2,†]

[1] *Forschungszentrum Jülich, Institute for Energy and Climate Research (IEK-STE), 52428 Jülich, Germany*
[2] *Institute for Theoretical Physics, University of Cologne, Köln, 50937, Germany*
[3] *Department of Mathematics, Massachusetts Institute of Technology, Cambridge, MA 02139, U.S.A.*
[4] *Physics Department, Williams College, 33 Lab Campus Drive, Williamstown, MA 01267, U.S.A.*

This Supplementary Material contains six Supplementary Notes and eight Supplementary Figures.

* f.kaiser@fz-juelich.de

† d.witthaut@fz-juelich.de

Supplementary Figures 3

Supplementary Notes 11
Supplementary Note 1: Minimising dissipation using Lagrange's method 11
Supplementary Note 2: Minimising dissipation using Karush-Kuhn-Tucker conditions 11
Supplementary Note 3: Proof of Theorem 1 13
Supplementary Note 4: Minimising the dissipation explicitly for the five- and three node network 14
Set of equations for five node network 14
Set of equations for three node network 16
Supplementary Note 5: Minimising cost using Karush-Kuhn-Tucker conditions 17
Supplementary Note 6: Identification of global minima of dissipation for tree networks using edge betweennees 18

Supplementary References 19

SUPPLEMENTARY FIGURES

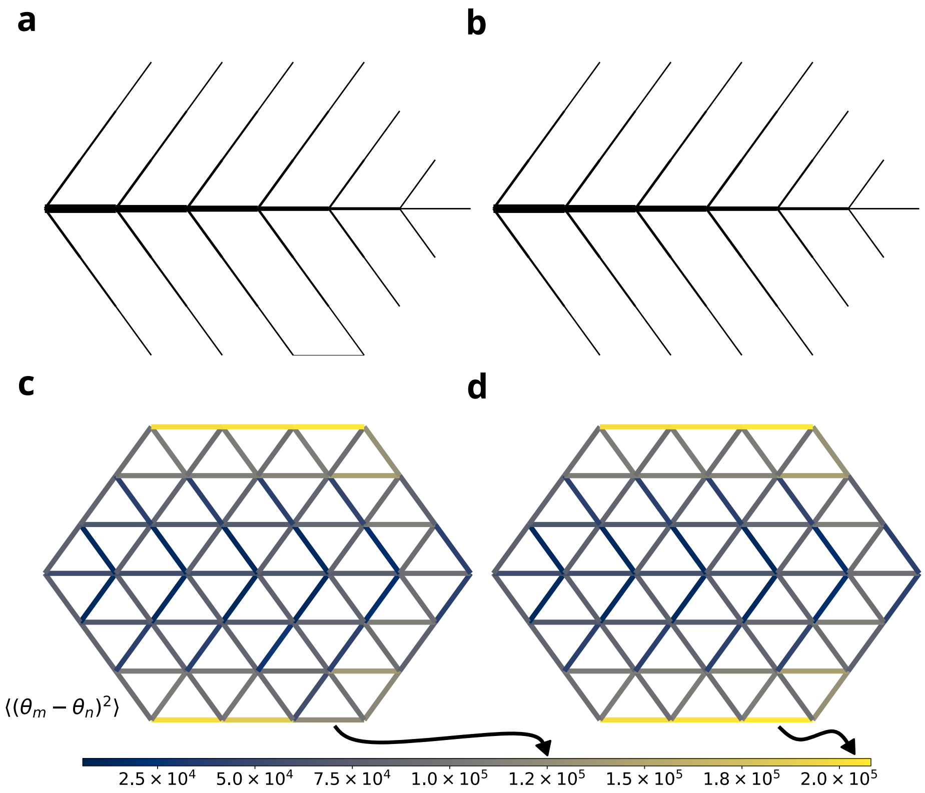

Supplementary Figure 1. **Adding a new loop significantly reduces the average squared potential drop along the corresponding vein. a,b** We allow for a loop to be added to a tree network whose capacities are optimised for the fluctuating sink model with cost parameter $\gamma = 0.84$, mean $\mu = -1$ and standard deviation $\sigma = 0.5$. **c,d** The loop formation significantly reduces the average squared potential drop along the corresponding edge (marked by arrows). The reduction is by approximately 42% of the original potential drop and thus provides significant stress relief for the network.

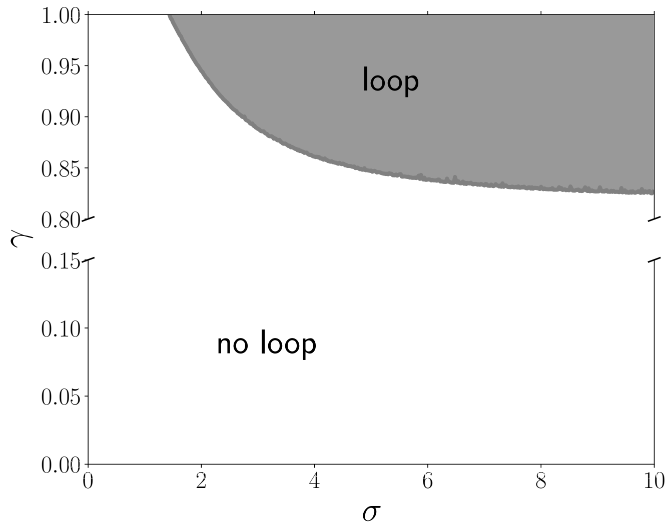

Supplementary Figure 2. **Phase diagram of the globally optimal network structure for the small five node network shown in Figure 2.** In the grey region in the upper right corner, loop formation is beneficial for the network dissipation. Interestingly, loop formation starts to be beneficial at a nonzero value of the fluctuation strength σ for any value of the cost parameter γ. Sinks have mean $\mu = -1$ in this setting.

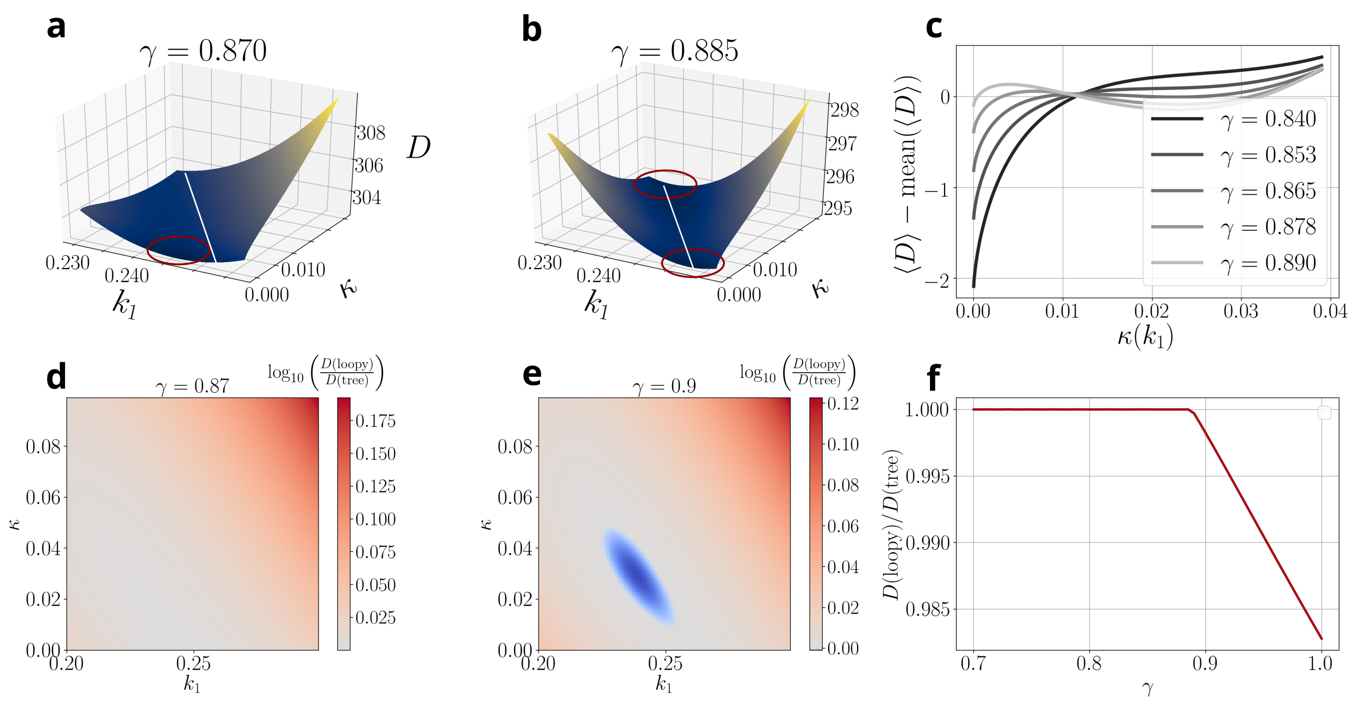

Supplementary Figure 3. **Emergence of loops for the five node network shown in Figure 2**. The landscape of dissipation for the loopy network has a single minimum for $\gamma = 0.87$ **a** as marked by a red circle. However, increasing the cost parameter to $\gamma = 0.885$, a second, local minimum emerges through a saddle node bifurcation **b**. Note that the second minimum is a local one for this set of parameters which turns into a global one for $\gamma > \gamma_c \approx 0.888$. **c** Moving along the white line indicated in panels (a) and (b) for varying values of γ, the emergence of a second, local minimum becomes visible. **d,e** We plot $\log_{10}(D_{\mathrm{loopy}}/D_{\mathrm{tree}})$ as a function of the edge capacities k_1 and $k_5 = \kappa$ in the loopy network. For $\gamma = 0.87$ we have $D_{\mathrm{loopy}} > D_{\mathrm{tree}}$ for all values of k_1 and κ. Hence the optimum network is a tree. (e) For $\gamma = 0.9$ there is a global minimum of D_{loopy} with $D_{\mathrm{loopy}} < D_{\mathrm{tree}}$ (blue). Hence, the optimum network is loopy. **f** The ratio of dissipations $D_{\mathrm{loopy}}/D_{\mathrm{tree}}$ for the optimum loopy and optimum tree networks as a function of the scaling exponent γ. This plot was produced for parameters $\sigma = 3$, $\mu = -1$ and $K = 1$.

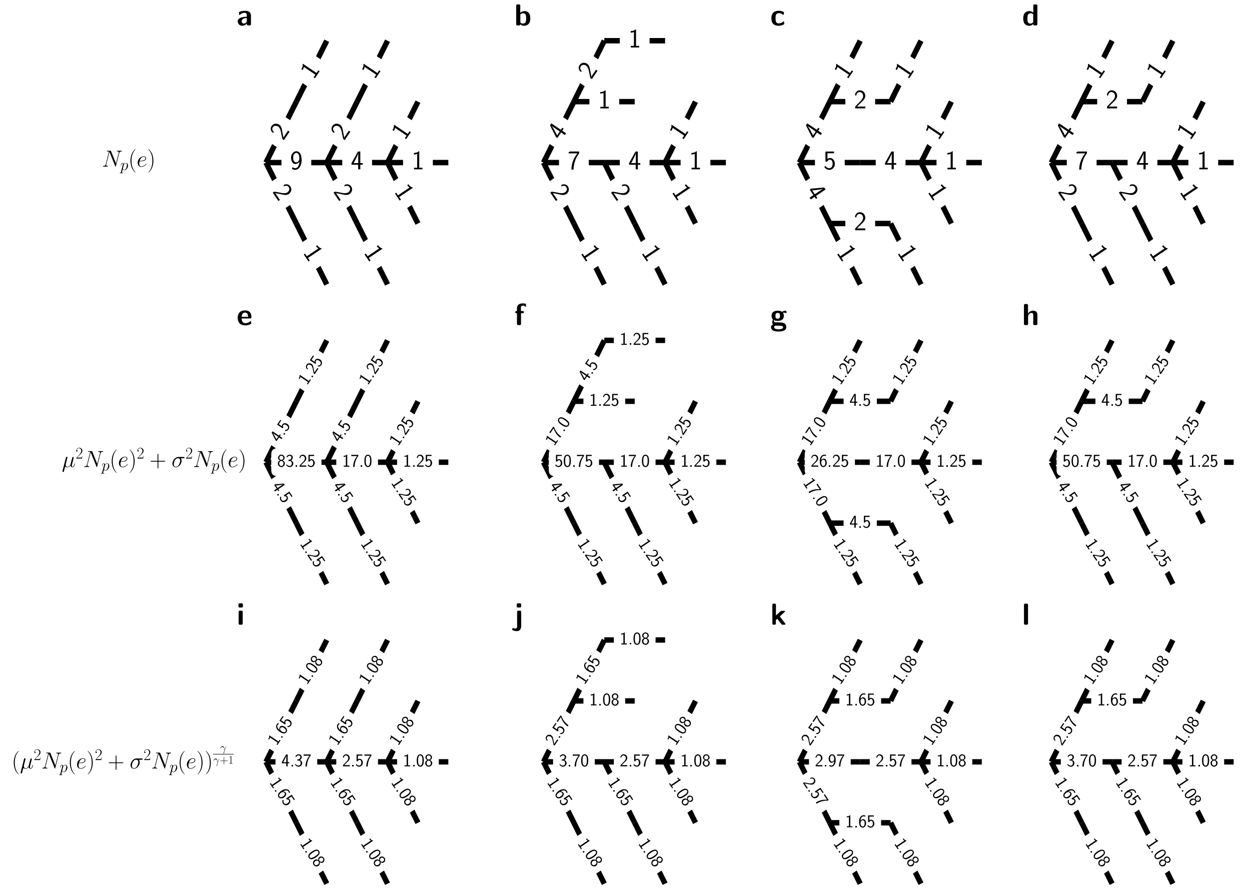

Supplementary Figure 4. **Edge betweenness may be used to identify the global minima of dissipation.** **a-d** Numbers on edges give the value of edge betweenness for different exemplary spanning trees on small triangular networks. **e-h** Measure of edge betweenness $N_p(e)\sigma^2 + N_p(e)^2\mu^2$ for mean of fluctuations $\mu = 1$ and standard deviation $\sigma = 0.5$. **i-l** Taking the measure of edge betweenness to the power of $\gamma/(\gamma+1)$ where here $\gamma = 0.5$ to calculate the contribution of an edge to the tree network dissipation, we can identify the globally minimising topology: we can conclude that the network shown in panels a and e is the globally optimal network, since it minimises the sum over all edges. This is due to the fact that the quasi-norm has a concave structure such that the minima are located at the boundaries of the domain: the network in panels (a,e,i) maximises the spread between the individual values of edge betweenness, accumulating as much flow as possible on as few edges as possible and thus minimises the dissipation.

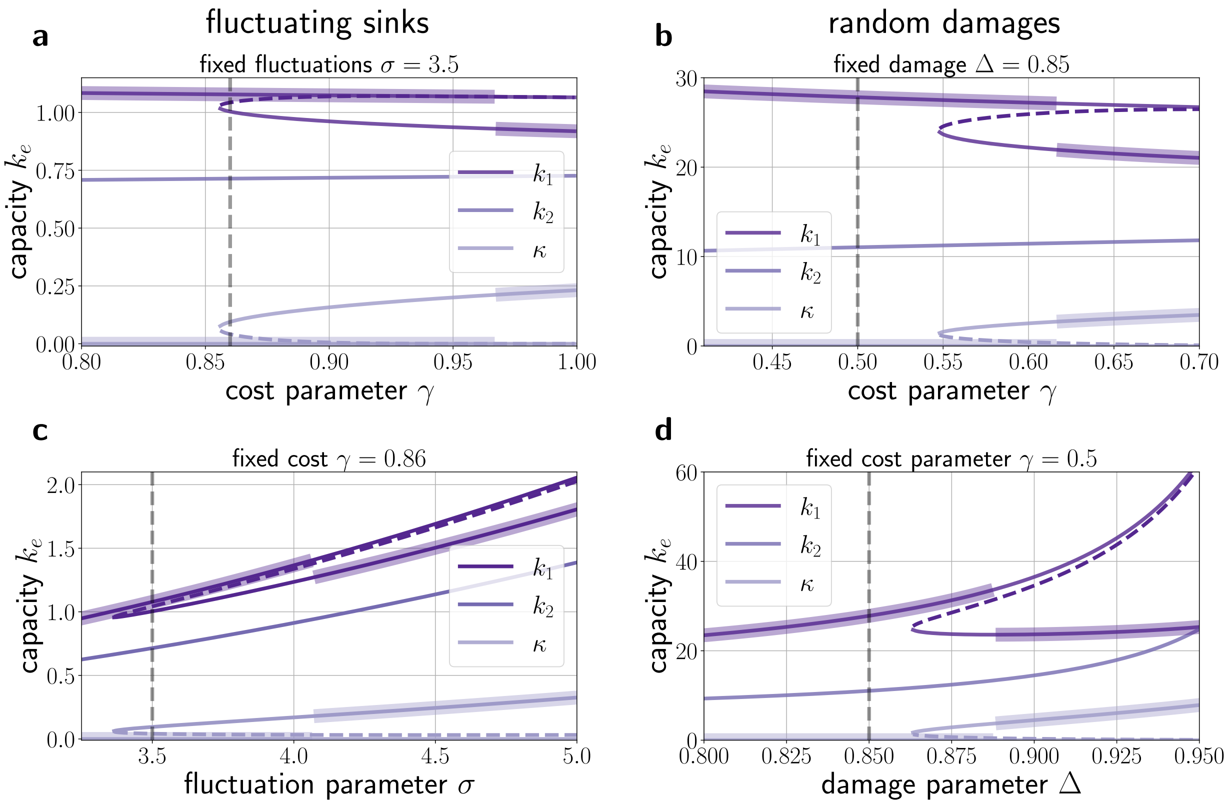

Supplementary Figure 5. **Discontinuous transition for minimum cost topologies** Capacities at the global minimum (thick lines) show a discontinuous transition to loop formation for different models for the five node network shown in Figure 2: Here, we minimise the cost while keeping the average network dissipation fixed at a given value D. **a,c** We analyse the edge capacities k_e at the local minima (straight lines) and saddle (dotted lines) for varying cost parameter γ (a) and varying fluctuation parameter σ (c) for the fluctuating sink model with fluctuation mean $\mu = -1$. For both parameters, the capacity at the loop κ (light purple) undergoes a saddle node bifurcation which causes a discontinuous transition in the global minima (thick lines) from non-loopy to loopy networks. **b,d** An analogous saddle-node bifurcation in the capacities k_e may be observed in the generalised damaged bond model in terms of both the cost parameter (b) and the damage parameter (d). The dissipation is fixed to $D = 90$ in panels (a) and (c) and to $D = 1$ in panels (b) and (d).

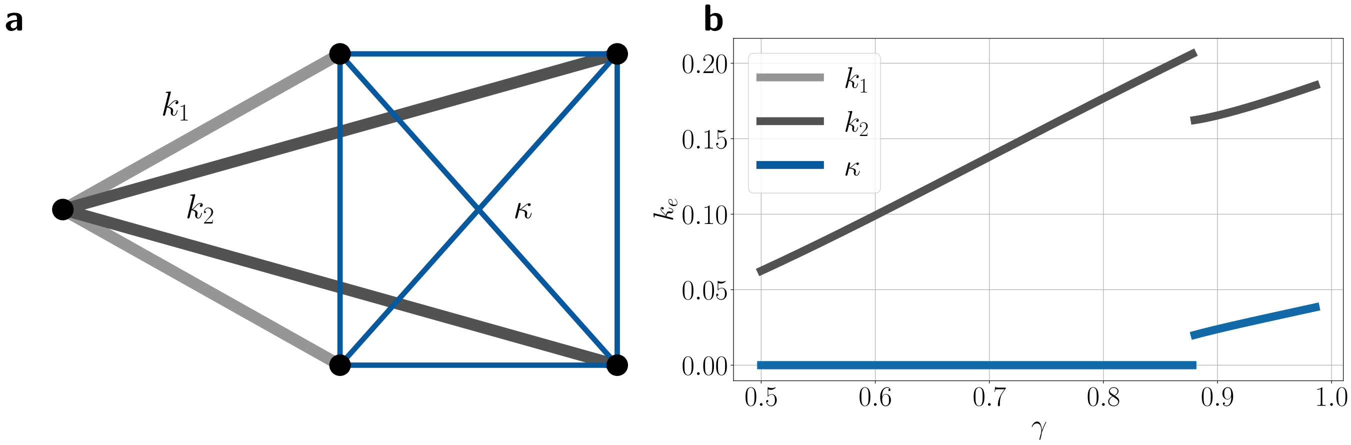

Supplementary Figure 6. **Discontinuous transition to loop formation in non-planar network a** To analyse the transition to loop formation in the simplest non-planar graph, the set of potential edges $\mathcal{E}$ is chosen as the edge set of the complete graph on five nodes K_5. Similar to the network setup shown in Figure 2a, we choose the node on the left as the node supplying the entire network and start from the tree network indicated by grey edges. **b** We then increase the cost parameter γ and perturb the edge capacities according to the procedure described in the methods section to monitor when new edges form. Again, we observe a discontinuous transition to loop formation. Due to the high degree of symmetry in the network, we observe that all potential loops form simultaneously with a non-zero capacity κ.

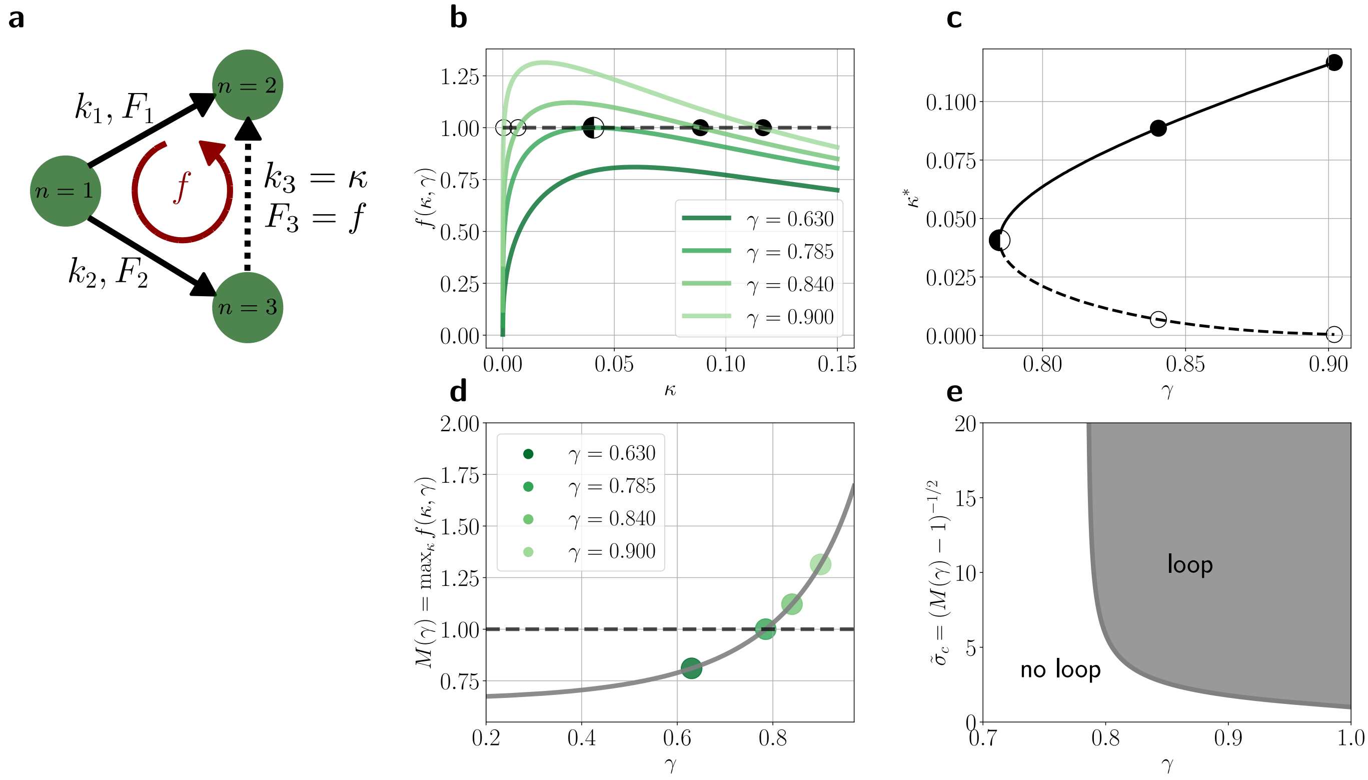

Supplementary Figure 7. **Three node network to analyse the transition to loop formation.** **a** Elementary network to study spontaneous loop formation in optimum supply networks. The network consists of three nodes (green circles) where node $n = 1$ has an inflow of two, $P_1 = 2$, and all other nodes have an outflow of unity. These in and outputs determine the flows $F_i, i \in \{1, 2, 3\}$ along the links with capacities k_i. The optimum topology for this set-up is a tree network. If the in- and outputs are fluctuating, however, an additional edge (dotted arrow) may be beneficial to reduce the average dissipation. This edge introduced a new degree of freedom expressed as a cycle flow f. **b** The points where the function $f(\kappa, \gamma)$ as defined in Eq. (6) crosses the dotted, black line at unity determine determine potential KKT points κ^*, where the loop in panel a is closed. The single peak crossing the dotted line clearly illustrates the saddle-node character of the bifurcation. **c** There are two potential KKT points κ^* which can be determined by finding the points where $f(\kappa, \gamma)$ is unity. One represents the saddle (empty circle) and the other one a minimum (filled circle). **d** Since the function shown in panel a has a single peak $M(\gamma)$ for these values of the cost parameter γ, the critical value γ_c where the loop starts to form can be determined by determining the value of γ for which this maximum reaches unity. **e** As a result of Eq. (7), we can find the critical value of the effective fluctuation strength $\sigma_c^2 = \sigma^2/\mu^2$ in dependence of the cost parameter γ by using the maximum $M(\gamma)$ and thus identify the region of the parameter space, where loop formation becomes beneficial.

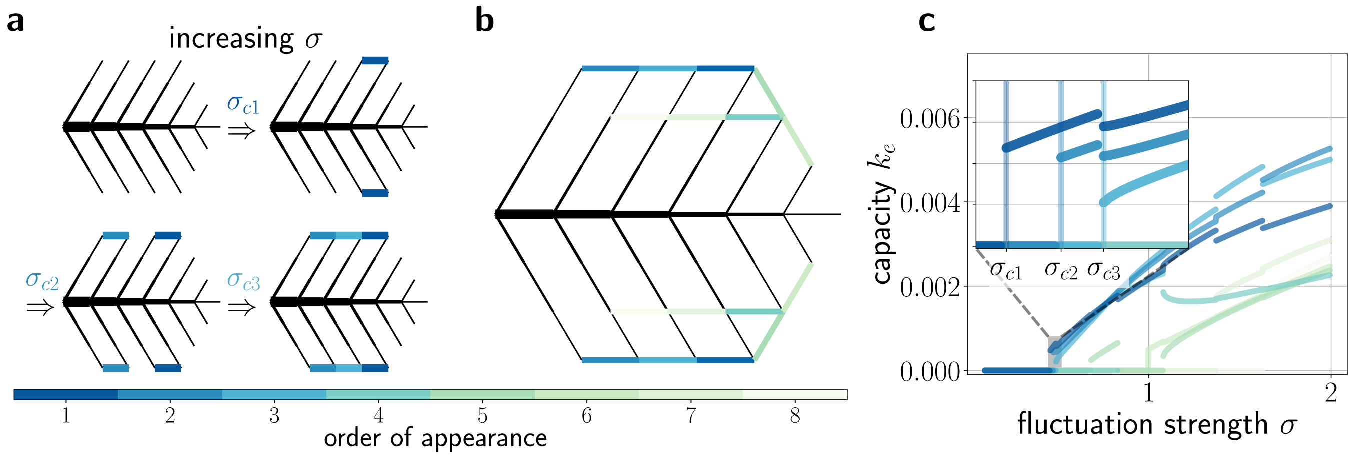

Supplementary Figure 8. **Discontinuous transition beyond the first loop for varying fluctuation strength σ a,b** We order the loops in a colour code according to their appearance with increasing fluctuation strength σ: the darker the edge colour, the earlier the edge appears. For the loop that appears as the i-th loop, we denote its fluctuation strength σ_c where the loop starts to become beneficial for the dissipation-optimised network. **c** The transition to loop formation is discontinuous beyond the first loop: loops appearing at higher values of σ again appear with a non-zero capacity as shown in detail in the inset. Cost parameter is fixed to $\gamma = 0.85$ for all panels.

SUPPLEMENTARY NOTES

Supplementary Note 1: Minimising dissipation using Lagrange's method

In this section, we illustrate how to minimise the dissipation with an equality constraint for a tree network using the method of Lagrange multipliers. The minimisation problem thus reads

$$\text{Minimise } \langle D_{\text{tree}}\rangle = \sum_{e\in E} \frac{\langle F_e^2\rangle}{k_e}$$
$$\text{subject to}$$
$$\sum_{e\in T} k_e^\gamma = K^\gamma.$$

This can be solved by minimising the Lagrange function

$$\mathcal{L}(k_e) = \sum_{e\in E} \frac{\langle F_e^2\rangle}{k_e} - \lambda\left(K^\gamma - \sum_{e\in E} k_e^\gamma\right).$$

The optimum edge weights are then found by taking the derivative and setting it to zero;

$$\frac{\partial \mathcal{L}}{\partial k_e} = -\frac{\langle F_e^2\rangle}{k_e^2} + \lambda\gamma k_e^{\gamma-1} \overset{!}{=} 0,$$
$$\Rightarrow k_e^* = \left(\frac{\langle F_e^2\rangle}{\lambda\gamma}\right)^{\frac{1}{1+\gamma}}.$$

Substituting this result into the capacity constraint yields the value of the Lagrange multiplier λ

$$\lambda = \frac{1}{\gamma}\left[\frac{\sum_{a\in E}(\langle F_a^2\rangle^{\frac{\gamma}{1+\gamma}}}{K^\gamma}\right]^{\frac{\gamma+1}{\gamma}}.$$

Then we finally obtain

$$k_e^* = \frac{(\langle F_e^2\rangle)^{\frac{1}{1+\gamma}}}{\left[\sum_{a\in E}(\langle F_a^2\rangle)^{\frac{\gamma}{1+\gamma}}\right]^{1/\gamma}} K.$$

This yields the minimised average dissipation

$$\langle D_{\text{tree}}^*\rangle = \frac{\left[\sum_{a=1}^{N-1}(\langle F_a^2\rangle)^{\frac{\gamma}{\gamma+1}}\right]^{(\gamma+1)/\gamma}}{K}.$$

Supplementary Note 2: Minimising dissipation using Karush-Kuhn-Tucker conditions

When minimising the dissipation in the tree network with a single additional edge, we have to take into account an inequality constraint $\kappa \geq 0$ for the newly added edge (m, n). We have the optimisation problem

$$\begin{aligned}
&\text{Minimise } \langle D_{\text{loopy}}\rangle = \sum_{e\in T} \frac{\langle F_e^2\rangle}{\tilde{k}_e} - \frac{\kappa}{1 + C_{m,n}\kappa} B_{m,n} \\
&\text{subject to} \\
&\sum_{e\in T} \tilde{k}_e^\gamma = K^\gamma - \kappa^\gamma, \\
&\kappa \geq 0.
\end{aligned} \tag{1}$$

This problem can be solved by using a generalisation of the method of Lagrange multipliers due to Karush, Kuhn and Tucker [1]. We first define the following Lagrange-type function

$$\begin{aligned}\tilde{\mathcal{L}}(\tilde{k}_e,\kappa) = &\sum_{e\in T}\frac{\langle F_e^2\rangle}{\tilde{k}_e} - \frac{\kappa}{1+C_{m,n}\kappa}B_{m,n}\\ &-\tilde{\lambda}\left(K^\gamma - \sum_{e\in T}\tilde{k}^\gamma - \kappa^\gamma\right) - \mu\kappa.\end{aligned}$$

Now we can proceed constructing the KKT conditions, whose solutions are potential local minima of the problem under the constraint and referred to as *KKT points*

$$\begin{aligned}\frac{\partial\tilde{\mathcal{L}}}{\partial\tilde{k}_e} &\overset{!}{=} 0, \quad \forall e\in T\cup\{\ell\}\\ K^\gamma &= \sum_{e\in T}\tilde{k}^\gamma + \kappa^\gamma,\\ -\kappa &\le 0\\ \mu &\ge 0,\\ \mu\kappa^* &= 0.\end{aligned}$$

We first compute the derivatives of the Lagrange function $\tilde{\mathcal{L}}$. In order to do so, we distinguish two types of edges: the ones that lie on the shortest path from the source to the new edge ℓ, i.e. where $e\in L\cup R$, and the other ones as sketched in Figure 2,b. For all edges $e\notin L,R$ that are not on the path we find

$$\begin{aligned}\frac{\partial\tilde{\mathcal{L}}}{\partial\tilde{k}_e} &= -\frac{\langle F_e^2\rangle}{\tilde{k}_e^2} + \tilde{\lambda}\gamma\tilde{k}_e^{\gamma-1} \overset{!}{=} 0,\\ \Rightarrow\tilde{k}_e &= \left(\frac{\langle F_e^2\rangle}{\tilde{\lambda}\gamma}\right)^{\frac{1}{1+\gamma}}.\end{aligned} \tag{2}$$

That is, we have the same result as in the tree except for a different normalisation factor $\tilde{\lambda}$. To get rid of the dependence of $\tilde{\lambda}$ on the edges not on the path $e\notin R,L$, we make use of the equality constraint

$$\frac{\partial\tilde{\mathcal{L}}}{\partial\tilde{\lambda}} \overset{!}{=} 0 \Rightarrow K^\gamma = \sum_{e\in T}\tilde{k}^\gamma + \kappa^\gamma.$$

Now summing over all edges that are not located on the path, we get

$$\begin{aligned}K^\gamma - \sum_{e\in L\cup R}\tilde{k}_e^\gamma - \kappa^\gamma &= \sum_{e\notin L\cup R}\tilde{k}_e^\gamma\\ &= \sum_{e\notin L\cup R}\left(\frac{\langle F_e^2\rangle}{\tilde{\lambda}\gamma}\right)^{\frac{\gamma}{1+\gamma}},\\ \Rightarrow\tilde{\lambda} &= \frac{1}{\gamma}\left[\frac{\sum_{e\notin L\cup R}(\langle F_e^2\rangle)^{\gamma/(\gamma+1)}}{K^\gamma - \sum_{e\in L\cup R}\tilde{k}_e^\gamma - \kappa^\gamma}\right]^{(\gamma+1)/\gamma},\end{aligned} \tag{3}$$

thus eliminating the entire dependence of $\tilde{\lambda}$ on $\tilde{k}_e, e\notin L\cup R$. For all edges on the path, $e\in L\cup R$, we find

$$\begin{aligned}\frac{\partial\tilde{\mathcal{L}}}{\partial\tilde{k}_e} &\overset{!}{=} 0\\ \Rightarrow\frac{\langle F_e^2\rangle}{\tilde{k}_e^2} &= -\frac{\kappa}{1+C_{m,n}\kappa}\frac{\partial B_{m,n}}{\partial\tilde{k}_e}\\ &\quad+\frac{\kappa^2 B_{m,n}}{(1+C_{m,n}\kappa)^2}\frac{\partial C_{m,n}}{\partial\tilde{k}_e} + \tilde{\lambda}\gamma\tilde{k}_e^{\gamma-1}.\end{aligned}$$

The derivatives appearing here can be readily calculated letting the edge $e \in L$, wlog;

$$\begin{aligned}
\frac{\partial C_{m,n}}{\partial \tilde{k}_e} &= -\frac{1}{\tilde{k}_e^2}, \\
\frac{\partial B_{m,n}}{\partial \tilde{k}_e} &= -2\frac{\langle F_e^2 \rangle}{\tilde{k}_e^3} \\
&\quad - \frac{1}{\tilde{k}_e^2} \sum_{a\in L, a\neq e} \frac{\langle F_e F_a \rangle}{\tilde{k}_a} + \frac{2}{\tilde{k}_e^2} \sum_{a \in R} \frac{\langle F_e F_a \rangle}{\tilde{k}_a}.
\end{aligned}$$

For the new edge $\ell = (m, n)$ we obtain

$$\begin{aligned}
\frac{\partial \tilde{\mathcal{L}}}{\partial \kappa} &= -\frac{B_{m,n}}{(1 + C_{m,n}\kappa)^2} + \tilde{\lambda}\gamma\kappa^{\gamma-1} - \mu \stackrel{!}{=} 0, \\
\Rightarrow \mu &= -\frac{B_{m,n}}{(1 + C_{m,n}\kappa)^2} + \tilde{\lambda}\gamma\kappa^{\gamma-1}.
\end{aligned}$$

The last KKT condition, the complementary slackness, thus yields

$$\begin{aligned}
\mu\kappa = 0 \; &\Rightarrow -\frac{\kappa B_{m,n}}{(1 + C_{m,n}\kappa)^2} + \tilde{\lambda}\gamma\kappa^{\gamma} \stackrel{!}{=} 0, \\
&\Rightarrow B_{m,n} = (1 + C_{m,n}\kappa)^2 \kappa^{\gamma-1}\gamma\tilde{\lambda} \; \vee \kappa = 0.
\end{aligned}$$

Note that $B_{m,n}$, $C_{m,n}$ and $\tilde{\lambda}$ depend on the updated capacities $\tilde{k}_e$. In general, i.e. for arbitrary network topologies and arbitrary parameters γ, σ and μ, the resulting set of equations does not have an analytical solution. However, it may be used to gain insight into the general structure of the minima as we will see in the next section.

Supplementary Note 3: Proof of Theorem 1

In this section we prove Theorem 1.

Proof. To prove this, we will make use of the last KKT condition, the complementary slackness in Eq. (19) in the main text

$$B_{m,n} = (1 + C_{m,n}\kappa)^2 \kappa^{\gamma-1}\gamma\tilde{\lambda}. \tag{4}$$

Now let $1 \gg \varepsilon > 0$ be a small positive number and let the loop capacity be $\kappa = \varepsilon$. We will now demonstrate that this capacity cannot be arbitrarily close to zero, i.e. that

$$\exists\; C \in \mathbb{R}^{>0} \text{ s.t. } \kappa = \varepsilon \geq C.$$

Let $\tilde{k}_{\min} = \min_{e\in L\cup R} \tilde{k}_e > 0$ be the minimum optimal capacity after the addition of the loop with capacity $\kappa = \varepsilon$ which can be obtained by solving the KKT problem resulting from Eq. (1). Now we can bound $B_{m,n}$ from above by

$$\begin{aligned}
\infty > B_{m,n}^{\max} &= \tilde{k}_{\min}^{-2} \left\langle \left(\sum_{e\in L} F_e - \sum_{e \in R} F_e \right)^2 \right\rangle \\
\geq B_{m,n} &= \left\langle \left(\sum_{e\in L} \frac{F_e}{\tilde{k}_e} - \sum_{e \in R} \frac{F_e}{\tilde{k}_e} \right)^2 \right\rangle.
\end{aligned}$$

On the other hand, we know that the first term on the right-hand side of Eq. (4) is larger than one $(1+C_{m,n}\kappa)^2 > 1$ since both expressions are positive, $C_{m,n} > 0$ and $\kappa > 0$, such that we can bound the entire right-hand side from below by

$$\kappa^{\gamma-1}\tilde{\lambda}\gamma < \kappa^{\gamma-1}\tilde{\lambda}\gamma(1 + C_{m,n}\kappa)^2.$$

Finally, we can also upper bound the Lagrange multiplier $\tilde{\lambda}$. To achieve this, we make use of the expression in Eq. (2) and Eq. (3) which relate the multiplier to the capacity of an edge $e \notin R \cup L$ that is not located on the shortest path connecting source and added edge. First we note that all edges have to have capacities smaller than the overall capacity $\tilde{k}_e < K$ by virtue of the capacity constraint. Thus we find

$$\tilde{\lambda} = \frac{1}{\gamma}\left[\frac{\sum_{e\notin L\cup R}(\langle F_e^2\rangle)^{\gamma/(\gamma+1)}}{\sum_{e\notin L\cup R}\tilde{k}_e^{\gamma}}\right]^{(\gamma+1)/\gamma},$$
$$>\tilde{\lambda}^{\min} = \frac{1}{\gamma K^{\gamma+1}}\left[\sum_{e\notin L\cup R}\langle F_e^2\rangle^{\gamma/(\gamma+1)}\right]^{(\gamma+1)/\gamma},$$
$$\Rightarrow \frac{1}{\tilde{\lambda}} < \frac{1}{\tilde{\lambda}^{\min}}.$$

Thus, upper bounding the left-hand side and lower bounding the right-hand side of Equation 4, we arrive at

$$\kappa^{\gamma-1}\tilde{\lambda} < \frac{B_{m,n}^{\max}}{\gamma},$$
$$\Rightarrow \kappa^{\gamma-1} < \frac{B_{m,n}^{\max}}{\gamma\tilde{\lambda}^{\min}},$$
$$\Rightarrow \kappa > \left(\frac{B_{m,n}^{\max}}{\gamma\tilde{\lambda}^{\min}}\right)^{1/(\gamma-1)} := C.$$

The last step holds due to the fact that $\gamma \in (0,1)$ such that the last manipulation results in the reciprocal on both sides of the inequality. Therefore we demonstrated that the optimal capacity of the loop κ has to be larger than the threshold parameter C if it is non-vanishing. □

Supplementary Note 4: Minimising the dissipation explicitly for the five- and three node network

Set of equations for five node network

Tree network Here, we explicitly derive the optima topology for the network shown in Figure 2,a. The network consists of four variable sinks at nodes 2, 3, 4, 5 (circles) and four edges (arrows) connecting them with capacities k_i and flows $F_i, i \in \{1, 2, 3, 4\}$. A fifth, potential edge is shown as dotted arrow. If it exists, it carries flow $\tilde{F}_5$ and has capacity κ. We first consider the case of a tree network, i.e. $\kappa = 0$.

In the fluctuating sink model, we describe the sinks as i.i.d. Gaussian random variables,

$$P_{2,3,4,5} \sim \mathcal{N}(\mu, \sigma).$$

The source at node 1 balances the sinks

$$P_1 = -\sum_{j=2}^{5} P_j.$$

While generic local minima with asymmetric capacities k_e exist, networks close to the global minimum generally show a high degree of symmetry [2, 3]. Therefore, in the following we consider symmetric optimal networks with capacities $k_3 = k_1$ and $k_4 = k_2$. If there is no loop ($\kappa = 0$), the flows may be calculated directly using the continuity equation (2)

$$F_1 = -(P_2 + P_4), \qquad F_2 = -P_4,$$
$$F_3 = -(P_3 + P_5), \qquad F_4 = -P_5,$$

which results in the following expressions for the second moments of flows

$$\langle F_1^2\rangle = \langle F_3^2\rangle = 4\mu^2 + 2\sigma^2, \quad \langle F_2^2\rangle = \langle F_4^2\rangle = \mu^2 + \sigma^2,$$
$$\langle F_1 F_3\rangle = 4\mu^2,\ \langle F_2 F_4\rangle = \mu^2,$$
$$\langle F_1 F_2\rangle = \langle F_3 F_4\rangle = 2\mu^2 + \sigma^2.$$

Using the optimal capacities and this set of equations, we can deduce explicit equations for the optimal capacities and thus the dissipation (see first section of this SI)

$$\langle D_{\text{tree}}\rangle = \frac{\left[2(\sigma^2+\mu^2)^{\frac{\gamma}{\gamma+1}} + 2(2\sigma^2+4\mu^2)^{\frac{\gamma}{\gamma+1}}\right]^{(\gamma+1)/\gamma}}{K}.$$

Loopy network How does this result change if we allow to close the loop as illustrated in Figure 2a, i.e., if we include the corresponding edge in the set of potential edges $\mathcal{E}$?

Let us assume a non-zero capacity $k_5 = \kappa > 0$ with flow $\tilde{F}_5$. In the following, we denote the flows and capacities in the loopy network with a tilde. In the presence of a loop, we can no longer determine the flows using the continuity equation alone.We can exploit Kirchhoff's voltage law Eq. (5)to eliminate the additional degree of freedom: it is given by a cycle flow f around the newly formed edge such that

$$\begin{aligned}
&\tilde{F}_1 = F_1 - f, \qquad &&\tilde{F}_2 = F_2,\\
&\tilde{F}_3 = F_3 + f, \qquad &&\tilde{F}_4 = F_4,\\
&\tilde{F}_5 = f
\end{aligned}$$

Using Kirchhoff's voltage law (Eq. (5)) we can express the cycle flow in terms of the remaining flows as

$$\begin{aligned}
&\frac{f}{\kappa} + \frac{\tilde{F}_3}{\tilde{k}_1} - \frac{\tilde{F}_1}{\tilde{k}_1} = 0,\\
\Leftrightarrow\quad & f = \left(2 + \frac{\tilde{k}_1}{\kappa}\right)^{-1} \times (F_1 - F_3)\,.
\end{aligned}$$

Finally, we can use this result to calculate the second moments of the flows as

$$\begin{aligned}
\langle \tilde{F}_1^2\rangle &= \langle \tilde{F}_3^2\rangle = 4\mu^2 + 2\sigma^2\left[1 - \frac{2\kappa}{2\kappa+\tilde{k}_1} + \frac{2\kappa^2}{(2\kappa+\tilde{k}_1)^2}\right]\\
\langle \tilde{F}_2^2\rangle &= \langle \tilde{F}_4^2\rangle = \mu^2+\sigma^2,\\
\langle \tilde{F}_5^2\rangle &= \langle f^2\rangle = 4\sigma^2\left(2+\frac{\tilde{k}_1}{\kappa}\right)^{-2}.
\end{aligned}$$

Using the KKT conditions, we can derive a closed-form solution for the loopy capacity κ. For the five node network, the dissipation of the loopy network reads

$$\langle D_{\text{loopy}}\rangle = \frac{2(\sigma^2+\mu^2)}{\tilde{k}_2} + \frac{2(4\mu^2+2\sigma^2)}{\tilde{k}_1} - \frac{4\sigma^2\kappa}{\tilde{k}_1^2 + 2\kappa\tilde{k}_1}.$$

Here, we made use of the symmetries in the network such that $\tilde{k}_1 = \tilde{k}_3$ and $\tilde{k}_2 = \tilde{k}_4$ and we used that in the situation at hand the two constants appearing in the expression are given by $B_{m,n} = \frac{4\sigma^2}{\tilde{k}_1^2}$ and $C_{m,n} = 2/\tilde{k}_1$. Taking the derivatives of the Lagrange function and setting them to zero, we arrive at the following set of equations

$$\begin{aligned}
&\tilde{k}_1 = \tilde{k}_3,\\
&\tilde{k}_2 = \tilde{k}_4 = \left(\frac{\mu^2+\sigma^2}{\tilde{\lambda}\gamma}\right)^{1/(1+\gamma)},\\
\Rightarrow &\tilde{\lambda} = \frac{(\mu^2+\sigma^2)}{\gamma}\left[\frac{2}{K^\gamma - 2\tilde{k}_1^\gamma - \kappa^\gamma}\right]^{\frac{\gamma+1}{\gamma}},\\
&\frac{4\sigma^2}{\tilde{k}_1^2} = (1+2\kappa/\tilde{k}_1)^2\kappa^{\gamma-1}\gamma\tilde{\lambda} \vee \kappa = 0,\\
&\gamma\tilde{\lambda}\tilde{k}_1^{\gamma+1} = (4\mu^2+2\sigma^2) - \frac{4\sigma^2\kappa(\kappa+\tilde{k}_1)}{(\tilde{k}_1+2\kappa)^2}.
\end{aligned}$$

From this expression, we can also immediately read off the Lagrange multiplier representing the inequality constraint in the case where $\kappa \neq 0$;

$$\mu = \frac{4\sigma^2}{\tilde{k}_1^2} - (1 + 2\kappa/\tilde{k}_1)^2 \kappa^{\gamma-1} \left[\frac{2(\mu^2+\sigma^2)^{\frac{\gamma}{\gamma+1}}}{K^\gamma - 2\tilde{k}_1^\gamma - \kappa^\gamma} \right]^{\frac{\gamma+1}{\gamma}} . \quad (5)$$

Inserting the expression for the Lagrange multiplier $\tilde{\lambda}$ into the last two equations, we arrive at the following set of equations for the two variables κ and $\tilde{k}_1$;

$$\begin{aligned} &(1 + 2\kappa/\tilde{k}_1)^2 \kappa^{\gamma-1} (\mu^2+\sigma^2) \left[\frac{2}{K^\gamma - 2\tilde{k}_1^\gamma - \kappa^\gamma} \right]^{(\gamma+1)/\gamma} \\ &= \frac{4\sigma^2}{\tilde{k}_1^2} \vee \kappa = 0, \\ &(\mu^2+\sigma^2) \left[\frac{2}{K^\gamma - 2\tilde{k}_1^\gamma - \kappa^\gamma} \right]^{\frac{\gamma+1}{\gamma}} \tilde{k}_1^{\gamma+1} \\ &= (4\mu^2 + 2\sigma^2) - \frac{8\sigma^2\kappa}{\tilde{k}_1 + 2\kappa} + \frac{4\sigma^2\kappa^2}{(\tilde{k}_1 + 2\kappa)^2}. \end{aligned}$$

However, these equations are still hard to solve analytically for arbitrary parameters μ, γ and σ.

Set of equations for three node network

We now turn to an even simpler network to shed further light on the transition to loop formation. In Figure 7a, we present a simple three node network with a potential loop with capacity κ and cycle flow $\tilde{F}_3 = f$.
The average dissipation in the presence of the loop can be readily calculated using the expression in Eq. 1 and is given by

$$\langle D_{\text{loopy}} \rangle = 2\frac{\sigma^2 + \mu^2}{\tilde{k}_1} - \frac{2\kappa\sigma^2}{\tilde{k}_1^2 + 2\kappa\tilde{k}_1}$$

The KKT conditions for this problem thus read

$$\begin{aligned} 2\lambda\gamma\tilde{k}_1^{\gamma+1} &= 2(\sigma^2+\mu^2) - \frac{4\sigma^2\kappa(\kappa + \tilde{k}_1)}{(\tilde{k}_1 + 2\kappa)^2} \\ 1 &= 2\tilde{k}_1^\gamma + \kappa^\gamma \\ \lambda\gamma\kappa^{\gamma-1}(2\kappa + \tilde{k}_1)^2 &= 2\sigma^2 \vee \kappa = 0 \end{aligned}$$

Putting everything together, we arrive at the following self-consistent equation for the loopy capacity κ

$$1 + \frac{\mu^2}{\sigma^2} = \frac{2\kappa\left(\kappa + \left(\frac{1}{2}(1-\kappa^\gamma)\right)^{1/\gamma}\right)}{\left(2\kappa + \left(\frac{1}{2}(1-\kappa^\gamma)\right)^{1/\gamma}\right)^2} + \frac{2\left(\frac{1}{2}(1-\kappa^\gamma)\right)^{(\gamma+1)/\gamma}\kappa^{1-\gamma}}{\left(2\kappa + \left(\frac{1}{2}(1-\kappa^\gamma)\right)^{1/\gamma}\right)^2}.$$

Note that the critical value of γ saturates for high values of σ^2 as shown in Figure 2. We can thus try to calculate this critical, saturated value γ_c by setting $\mu = -1$, w.l.o.g., and letting $\sigma^2 \to \infty$ to arrive at a self-consistent equation involving only γ and κ

$$1 = \frac{2\kappa\left(\kappa + \left(\frac{1}{2}(1-\kappa^\gamma)\right)^{1/\gamma}\right) + 2\left(\frac{1}{2}(1-\kappa^\gamma)\right)^{(\gamma+1)/\gamma}\kappa^{1-\gamma}}{\left(2\kappa + \left(\frac{1}{2}(1-\kappa^\gamma)\right)^{1/\gamma}\right)^2}$$

Now we want to find for which value of γ there is a $\kappa > 0$ that solves the above equation. To this end, we define the function

$$f(\kappa,\gamma) = \frac{2\kappa\left(\kappa + \left(\frac{1}{2}(1-\kappa^\gamma)\right)^{1/\gamma}\right) + 2\left(\frac{1}{2}(1-\kappa^\gamma)\right)^{1+1/\gamma}\kappa^{1-\gamma}}{\left(2\kappa + \left(\frac{1}{2}(1-\kappa^\gamma)\right)^{1/\gamma}\right)^2} \tag{6}$$

and plot it for different values of κ and γ in Figure 7,b. Thus, the critical value of the cost parameter where the loop starts to form, γ_c, can be calculated by finding the minimum value of the function where it approaches unity

$$\gamma_c = \min\left(\{\gamma \in (0,1) | \exists \kappa \in (0,1) \text{ s.t. } f(\kappa,\gamma) = 1\}\right).$$

Looking at the graph of $f(\kappa,\gamma)$ in Figure 7,b, we see that the function has a single maximum in terms of κ for a given value of γ. We can thus calculate the critical value γ_c by determining, for which value this maximum reaches unity for the first time. Denote by $M(\gamma) = \max_{\kappa\in(0,1)} f(\kappa,\gamma=\gamma)$ the maximum of the function for a given value of γ. Then we can also find the critical value γ_c as

$$M(\gamma_c) = 1.$$

We now turn back to non-infinite values of the fluctuation strength to map out the relationship between the critical fluctuation amplitude σ_c and the critical value of the cost parameter γ_c, similar to the phase diagram shown in Figure 2. For non-infinite values of the fluctuation strength σ, the above equation becomes

$$\begin{aligned} M(\gamma) &= 1 + \frac{\mu^2}{\sigma^2} \\ \Rightarrow \tilde{\sigma}_c &= \frac{1}{\sqrt{(M(\gamma)-1)}} \end{aligned} \tag{7}$$

where we defined the rescaled fluctuation strength $\tilde{\sigma} = \sigma/\mu$. Finally, we can also calculate the minimum rescaled fluctuation strength necessary for a loop to exist. To this end, we calculate $\lim_{\gamma\to 1} M(\gamma)$ which may be calculated analytically and note that the maximum $M(\gamma)$ strictly increases with the cost parameter γ

$$\begin{aligned} \lim_{\gamma\to 1} M(\gamma) &= \max_{\kappa\in(0,1)} \lim_{\gamma\to 1} f(\kappa,\gamma) = \max_{\kappa\in(0,1)}\left[\frac{6\kappa^2+2}{(3\kappa+1)^2}\right] \\ \Rightarrow \frac{\partial f(\kappa,\gamma=1)}{\partial\kappa} &= \frac{12(\kappa-1)}{(1+3\kappa)^3} < 0 \quad \forall \kappa \in (0,1). \end{aligned}$$

The derivative is strictly decreasing for the given interval, the maximum thus occurs at the boundary of the domain. The value at $\lim_{\kappa\to 0} f(\kappa,\gamma=1)$ is given by $M(\gamma) = 2$. We can thus determine the minimum necessary fluctuation strength for the loop to exist as

$$\sigma_{c,\min} = \frac{1}{\sqrt{2-1}} = 1.$$

Supplementary Note 5: Minimising cost using Karush-Kuhn-Tucker conditions

Instead of finding the capacities that minimise the dissipation for a given system cost, we can consider a dual problem: Minimising the system cost while fixing the average dissipation. Again, we want to find out when the first loop emerges. So denote by D the fixed, average dissipation and by C the cost. Formulating the KKT conditions for this related problem, we are thus left with the following optimisation problem

$$\begin{aligned} &\text{Minimise } C = \sum_{e\in T}\tilde{k}_e^\gamma + \kappa^\gamma \sum_{e\in T}\tilde{k}_e^\gamma \\ &\text{subject to} \\ &D = \frac{\langle F_e^2\rangle}{\tilde{k}_e} - \frac{\kappa}{1+C_{m,n}\kappa}B_{m,n} \\ &\kappa \geq 0. \end{aligned} \tag{8}$$

We again define the following Lagrange-type function

$$\tilde{\mathcal{L}}(\tilde{k}_e, \kappa) = \sum_{e\in T} \tilde{k}_e^{\gamma} + \kappa^{\gamma}$$
$$- \tilde{\lambda} \left(\sum_{e\in T} \frac{\langle F_e^2 \rangle}{\tilde{k}_e} - \frac{\kappa}{1 + C_{m,n}\kappa} B_{m,n} - D \right) - \mu\kappa.$$

Similarly, we can now identify the *KKT points* via the following set of equations

$$\frac{\partial \tilde{\mathcal{L}}}{\partial \tilde{k}_e} \stackrel{!}{=} 0, \quad \forall e \in T \cup \{\ell\}$$
$$D = \sum_{e\in T} \frac{\langle F_e^2 \rangle}{\tilde{k}_e} - \frac{\kappa}{1 + C_{m,n}\kappa} B_{m,n},$$
$$-\kappa \leq 0$$
$$\mu \geq 0,$$
$$\mu\kappa^* = 0.$$

Supplementary Note 6: Identification of global minima of dissipation for tree networks using edge betweennees

Suppose we assign a vector $\boldsymbol{v}(T) \in \mathbb{R}^{|\mathcal{E}|}$ to each tree T, where the entry at position i of edge e corresponds to the measure of edge betweenness $N_p(e)\sigma^2 + N_p(e)^2\mu^2$. These vectors will in general contain mainly zero entries for tree networks. In this case, finding the globally optimal tree network corresponds to finding the vector $\boldsymbol{v}(T)$ that is globally minimal with respect to the quasi-norm induced by $\frac{\gamma}{\gamma+1} \in [0, 0.5]$, i.e. the tree T^* for which $\|\boldsymbol{v}(T^*)\|_{\frac{\gamma}{\gamma+1}}$ is minimal. Note that the ratio of cost parameters $\frac{\gamma}{\gamma+1} < 1$ is smaller than one, such that it does not induce a proper L^p-norm.

For the topology used throughout this paper, i.e. the network constructed by cutting out edges and nodes of a triangular grid such that it corresponds to the shape of a leaf (see Figure 2c), we can demonstrate how to find the global minimum. In Figure 4, we show different realizations of spanning trees for a small sample network and the according edge betweenness as numbers on the edges which can then be used to form the vectors $\boldsymbol{v}(T)$. In panels (e-h), we compare the entries of this vector for the spanning trees. Notably, the network shown in panels (a,e) displays the highest allocation of edge betweenness on as few links as possible and thus the highest imbalance in the vectors $\boldsymbol{v}(T)$. Indeed, taking the entries to the power of $\gamma/(\gamma+1)$ in order to calculate each edge's contribution to the dissipation (panels i-l), we observe that the tree with the highest allocation has the minimum dissipation. This can easily be seen by adding up the values on the three edges connected to the source in the four panels and noting that the other values remain the same, even if distributed among the edges differently.

We also numerically tested this argument by randomly sampling the local tree minima, but also the shortest path trees for larger networks and did not find any network with less dissipation.

SUPPLEMENTARY REFERENCES

[1] Kuhn, H. W. & Tucker, A. W. Nonlinear programming. In *Proceedings of the Second Berkeley Symposium on Mathematical Statistics and Probability*, 481–492 (University of California Press, Berkeley, Calif., 1951).

[2] Katifori, E., Szöllősi, G. J. & Magnasco, M. O. Damage and fluctuations induce loops in optimal transport networks. *Phys. Rev. Lett.* **104**, 048704 (2010).

[3] Ronellenfitsch, H. & Katifori, E. Global Optimization, Local Adaptation, and the Role of Growth in Distribution Networks. *Phys. Rev. Lett.* **117**, 138301 (2016).
