# Supplementary material for: Discontinuous transition to loop formation in optimal supply networks
Source: Nat Commun. 2020 Nov 16;11:5796. doi: 10.1038/s41467-020-19567-2 (PMC7670464; doi:10.1038/s41467-020-19567-2)
Supplement: Supplementary file 2 — Peer Review File [file 41467_2020_19567_MOESM2_ESM.pdf]

Reviewer #1 (Remarks to the Author):

The manuscript studies loop formation in "optimal" supply networks as a broad question including when and where loops form. Specifically, the context of loop formation in tree networks when minimizing average dissipation for edge-damage and fluctuating sink models. The mathematics show, among other things, that cycles form through saddle-node bifurcations and that the loops appear discontinuously when decreasing edge costs for global minimum dissipation. A relationship to betweenness is also outlined and used to find global optima of network dissipation. The work is quite interesting and the results are insightful and novel, while also being applicable to a broad interpretation of "supply network".

I do have a number of comments:
1. The introduction aims to outline the importance of research on loop formation in supply networks by describing a trade-off between efficiency and resilience, but very limited evidence or references are given to support this claim. Specifically, up to date references focusing on supply network design given different measures of topological resilience would be useful, for instance. Moreover, an elaboration concerning the broader implications of how their results contribute to the discussion of the efficiency-resilience trade-off would further highlight the significance of their results. Likewise, in the discussion it would be very useful to address how the findings of the paper link back to some of the topics presented in the introduction. This would "round out" the paper storyline.

2. The use of the word "optimal" is confusing since there are many common interpretations in the supply chain/network setting that one could use, and hence should be clarified much earlier in the manuscript (and perhaps the title as well). Moreover, the motivation behind why this particular measure is selected is not clearly stated, nor is its meaning in the context of supply networks.

Also, concerning the reference to Bollobas's book: it does not seem to contain the definition either (perhaps I missed it, but please include a page number if so). The authors should elaborate and include better sources otherwise.

3. The presentation of the optimization could be improved by using a more typical/standard optimization form and organization. For instance, much of page 2 is confusing until equation 9 is finally discussed. A more standard optimization form could improve the readability to a broader audience.

Similarly, cost parameter γ is repeatedly used throughout the manuscript but there is no clear explanation for it. Although the two references do provide some details, it should be included in the text along with a brief explanation of its significance within the context of the particular questions being addressed in this research.

4. Results for the fluctuating sink and broken-bond models are currently in one section, but breaking this into separate sections versus repeated jumping between them throughout the manuscript should improve readability.

5. Figure 3f is very difficult to follow/interpret: a more descriptive caption is necessary. For instance, it is unclear why the line colors change. Moreover, the caption indicates "the darker the edge color, the later the edge appears" but in the legend darker colors correspond to lower value and that should mean the edge would appear earlier - unless perhaps I misread something. However, this color scheme is then reversed in Figure 4, which says "Color code corresponds to order of appearance of edges from dark (first) to light (last)"...this causes the reader confusion. Please clarify/standardize.

6. The experimental results are appropriate, and are intuitive given the mathematics.

7. I'm curious about potential rigorous prediction of when (with regard to the cost parameter) loop

edges form/discontinuous jumps occur. Currently, this is examined though correlation with pressure drops, but there may be analytical work for the toy model that can be incorporated, or at least commented on in the discussion.

Minor comments:
1. The function c(γ,e) is used in line 410 without any definition.

2. A few cases in the manuscript the authors write "see SI for details" without providing a specific section.

Reviewer #2 (Remarks to the Author):

The paper is clear and well written. Its main contribution is that it has explored the transition from branched to looped networks, by proposing a measure of stress that allows prediction of the location of new loops to close. I have two major comments, which come from my background of hydraulic engineer, and some minor comments.
Major comments
1 - Besides cost and resilience related consideration, laying a pipe in a hydraulic network has very practical significance. In fact, it is necessary for supplying water to the users located along the pipe. In hydraulic model, demands are allocated to end nodes only for modelling purposes, even if users are located along pipes (see paper "Using EPANET for modelling water distribution systems with users along the pipes"). In the field of water distribution network, branched networks hidden inside looped networks are searched for both modelling (e.g., papers "Comparison of newton-raphson global and loop algorithms for water distribution network resolution" and "Reformulated Co-Tree Flows Method Competitive with the Global Gradient Algorithm for Solving Water Distribution System Equations") and optimization (e.g., papers "The importance of the minimum path criterion in the design of water distribution networks" and "Fast network multi-objective design algorithm combined with an a posteriori procedure for reliability evaluation under various operational scenarios") purposes. Therefore, the tree network has only a theoretical significance in most cases.
2 - I could see that the Authors set up an optimization problem with dissipation as objective function to minimize and budget constraints. In practical problems, such as water network design, budget is the objective function to minimize while there is a constraint on dissipation. Indeed, budget is the critical issue for engineers, which is then considered as objective function to minimize. How can this aspect be considered in your approach? Please reply in the text.
Minor comments
There are some oversights in the text
3 - lines 15-17. Indeed the trade-off is between cost and resilience.
4 - line 21. "Establish"
5 - Equation 2. Why is there "!"?
6 - In text and equations, the Authors sometimes use "ke" and other times "k". What is the difference?
7 - line 329 "updated"

Reviewer #3 (Remarks to the Author):

I have reviewed the manuscript entitled “Discontinuous transition to loop formation in optimal supply networks,” written by Franz Kaiser et al. This paper studies an interesting question: how to form loops in supply network? The authors discovered a discontinuous transition, which is very interesting. Furthermore, the authors derived the analytical solution. Yet, I do have several concerns and

comments on the current form of the manuscript regarding the models, methods, and the results of this work. I am quite open to looking at a revised version if the author can address in a satisfactory fashion the issued discussed below:

In the abstract, it is unclear why adding new redundant edges is costly ye beneficial for resilience. How to define the resilience and cost of networks? There are many papers and also many definitions of these terms. I do not know what the definitions are here? More importantly, why is it important to study the effect of a single edge? As we know, the sizes of real-world networks are huge. The impact of a single link is almost nothing. Why should one care about the effect of a single edge on a huge network?

The discontinuous bifurcation is very interesting. The authors studied the phase transition from no loop state to a single loop state. As we know, many real networks are sparse, but they have loops. Why should one worry about this transition if one knows that the networks already have loops?

In Eq. (2), it is unclear what is the meaning of =^!. The authors give a reference to Eq. (3), but it is unclear why it is defined as this form. Eq. (4) is also confusing. The notations are hard to understand. For example, in Eq. (4), the authors use $F(n,m)$ that corresponds to an edge between node m and n. But in Eq.(1) and (2), the authors used $I_{n,e}$, where n denotes a node, and e means an edge. The authors also define some terms with an index of link, for example, k_e.

After Eq. (5), the authors described the F_e and $-F_e$. It is still confusing how to choose the sign of F_e. Does it depend on the sign of I or on where are the source and sink?

The authors obtain Eq. (7) from Eq. (3). Is there any assumption that k_e is the same? Otherwise, how to get Eq. (7)? The equation after Eq. (11) seems reasonable to me. Why is the resource constraint cast into Eq. (8)?

The statement in the first two sentences on page 3 is not true. Most of the real-world networks do not have geometric constrains. Is this tool only useful for the spatially embedded networks without any long-range links?

I cannot accept the current version of the paper.

Response to Reviewers for:

Discontinuous transition to loop formation in optimal supply networks

Franz Kaiser,[1,2,*] Henrik Ronellenfitsch,[3,4] and Dirk Witthaut[1,2,†]

[1]*Forschungszentrum Jülich, Institute for Energy and Climate Research (IEK-STE), 52428 Jülich, Germany*

[2]*Institute for Theoretical Physics, University of Cologne, Köln, 50937, Germany*

[3]*Department of Mathematics, Massachusetts Institute of Technology, Cambridge, MA 02139, U.S.A.*

[4]*Physics Department, Williams College, 33 Lab Campus Drive, Williamstown, MA 01267, U.S.A.*

[*] f.kaiser@fz-juelich.de

[†] d.witthaut@fz-juelich.de

REPLY TO REVIEWER 1

"The manuscript studies loop formation in "optimal" supply networks as a broad question including when and where loops form. Specifically, the context of loop formation in tree networks when minimizing average dissipation for edge-damage and fluctuating sink models. The mathematics show, among other things, that cycles form through saddle-node bifurcations and that the loops appear discontinuously when decreasing edge costs for global minimum dissipation. A relationship to betweenness is also outlined and used to find global optima of network dissipation. The work is quite interesting and the results are insightful and novel, while also being applicable to a broad interpretation of "supply network"."

→ We thank the reviewer for the positive assessment of our work and for taking the time to review our paper in depth. We are grateful for the helpful comments which we address below point by point.

Major comments

"1. The introduction aims to outline the importance of research on loop formation in supply networks by describing a trade-off between efficiency and resilience, but very limited evidence or references are given to support this claim. Specifically, up to date references focusing on supply network design given different measures of topological resilience would be useful, for instance. Moreover, an elaboration concerning the broader implications of how their results contribute to the discussion of the efficiency-resilience trade-off would further highlight the significance of their results. Likewise, in the discussion it would be very useful to address how the findings of the paper link back to some of the topics presented in the introduction. This would "round out" the paper storyline."

→ We thank the reviewer for this helpful comment. In the revised version of the manuscript, we substantially extended the introduction to elucidate the broader context of the trade-off between cost and resilience. In particular, we added several new references discussing the trade-off between cost and resilience throughout different disciplines.

In addition to that, we now clarify which notions of cost and resilience we apply in our

manuscript in the introduction:

"Here, we analyse the transition to loop formation on a theoretical basis and derive several analytical results. We consider optimal network structures in the sense that function is optimised while costs are constrained or vice versa. Two aspects of resilience are studied in detail – damage to edges and fluctuations of supply and demand. In particular, we investigate the optimal structure as a function of the severity of damage and the strength of fluctuations."

Finally, we significantly extended the discussion to now incorporate how our results and methods contribute to a topology-driven analysis of network resilience and to illustrate their relationship towards percolation-driven approaches to resilience.

"2. The use of the word "optimal" is confusing since there are many common interpretations in the supply chain/network setting that one could use, and hence should be clarified much earlier in the manuscript (and perhaps the title as well). Moreover, the motivation behind why this particular measure is selected is not clearly stated, nor is its meaning in the context of supply networks.
Also, concerning the reference to Bollobas's book: it does not seem to contain the definition either (perhaps I missed it, but please include a page number if so). The authors should elaborate and include better sources otherwise."

→ We thank the reviewer for making us aware of the unclear usage of the word "optimal". In the revised version of the manuscript, we clarified our notion of optimality in several regards. Firstly, we now discuss our approach to optimality in the introduction (see answer to the last question).

Furthermore, we now describe the measure of optimality in more detail when introducing the optimisation problem in the modelling section:

"We now illustrate how to determine the *optimal* supply network that is described by the above the set of equations. To this end, we want to find the edge capacities that determine the network structure that is optimal for performing a given task. Throughout this manuscript, we call the network structure *optimal* if the edge capacities are such that the overall network dissipation is minimised, as suggested for example in Refs. [1–3]."

Finally, we now discuss our results in detail in the broader context of other measures of

network resilience and optimality in the discussion section.

We also thank the reviewer for pinpointing us to the imprecise reference to the book by Bollobás. In the revised manuscript, we removed the reference to ”Thomson's principle” (which is contained in the book by Bollobás in Chapter IX, Theorem 1) to avoid potential conclusion with the corresponding optimisation problem and updated the reference to ”Kirchhoff's voltage law” to include the page number.

”3. The presentation of the optimization could be improved by using a more typical/standard optimization form and organization. For instance, much of page 2 is confusing until equation 9 is finally discussed. A more standard optimization form could improve the readability to a broader audience.
Similarly, cost parameter γ is repeatedly used throughout the manuscript but there is no clear explanation for it. Although the two references do provide some details, it should be included in the text along with a brief explanation of its significance within the context of the particular questions being addressed in this research.”

→ We thank the reviewer for pointing out that the presentation of the optimisation problem was unclear here. In the revised version of the manuscript, we separated the section discussing the model and the section discussing the optimisation problem to now clearly state which optimisation problem we seek to address in the manuscript. To this end, we also reformulated our presentation of the general set-up: we now introduce linear flow networks without referring to the underlying optimisation problem which is a result of ”Thomson's principle” and instead simply state the equations for linear flow networks to avoid potential confusion with the actual optimisation problem that we analyse.

In addition to that, we have added a discussion of the physical meaning of the cost parameter γ below Eq. (6).

”4. Results for the fluctuating sink and broken-bond models are currently in one section, but breaking this into separate sections versus repeated jumping between them throughout the manuscript should improve readability.”

→ In the revised version of the manuscript, we restructured the presentation of the optimization problem and the methodology. We now created separate subsections for the two models in both the modelling section and the results section and believe that this greatly improves the readability of the manuscript.

"5. Figure 3f is very difficult to follow/interpret: a more descriptive caption is necessary. For instance, it is unclear why the line colors change. Moreover, the caption indicates "the darker the edge color, the later the edge appears" but in the legend darker colors correspond to lower value and that should mean the edge would appear earlier - unless perhaps I misread something. However, this color scheme is then reversed in Figure 4, which says "Color code corresponds to order of appearance of edges from dark (first) to light (last)"...this causes the reader confusion. Please clarify/standardize."

→ We apologise for the ambiguity in the two figure legends. In fact, the legend in Figure 3 contained a mistake and should reference the same color ordering as in the old Figure 4. We decided to split the original Figure 3 into two separate Figures to address the order of appearance issue and to more clearly present the ideas contained in the original figure. In the new Figure 4, we included additional details on the appearance of the loops and explained the colour code. In addition to that, we added a new section to the manuscript to discuss the meaning of the new Figure 4 in more detail. We hope that this clarifies the issue raised by the reviewer.

"6. The experimental results are appropriate, and are intuitive given the mathematics."

→ We are happy that the reviewer finds that our numerical results fits the mathematics.

"7.. I'm curious about potential rigorous prediction of when (with regard to the cost parameter) loop edges form/discontinuous jumps occur. Currently, this is examined though correlation with pressure drops, but there may be analytical work for the toy model that can be incorporated, or at least commented on in the discussion."

→ We thank the reviewer for her/his interest in our work and for pointing us to the value

of a rigorous prediction of the discontinuity. In fact, this comment has led us to find another very interesting approximation of the critical cost parameter and the associated saddle-node bifurcation: We performed an additional theoretical analysis for an even simpler network containing only three nodes, since an analytical analysis of the optimisation problem is already very involved in the five node case (see Supplementary Note 4). For the three node network, we managed to get another estimate of the critical cost parameter γ_c where the loop starts to form which we now discuss in a separate Figure and section in the Supplementary Material. For this particular case, we were also able to derive a closed-form solution for the phase diagram in terms of fluctuation strength σ and cost parameter γ where the loop starts to become beneficial (see Fig. S7e). We refer to this new result in the revised manuscript after discussing our results for the five node network on page 4 as follows:

"In a separate Supplementary Figure S7, we illustrate the nature of this transition for an even simpler network and find a closed-form solution for the region of the parameter space where loop formation is beneficial."

Minor comments:

"1. The function $c(\gamma, e)$ is used in line 410 without any definition."

→ We revised the presentation of the constant $c(\gamma, e)$ such that the corresponding part of the manuscript now reads

"the capacities of the edges e along the shortest path from the source to the loop, $e \in R \cup L$, change only by a constant factor $c(\gamma, e)$, i.e. $\tilde{k}_e = c(\gamma, e)\, k_e$"

"2. A few cases in the manuscript the authors write "see SI for details" without providing a specific section. "

→ We thank the reviewer for pointing out this unclear reference to the SI. We now restructured the SI to include more clearly separable sections and updated the references to it throughout the manuscript.

REPLY TO REVIEWER 2

"The paper is clear and well written. Its main contribution is that it has explored the transition from branched to looped networks, by proposing a measure of stress that allows prediction of the location of new loops to close. I have two major comments, which come from my background of hydraulic engineer, and some minor comments."

→ We thank the reviewer for this positive assessment of our work and are grateful for the helpful comments which we address below point by point.

Major comments

"1 - Besides cost and resilience related consideration, laying a pipe in a hydraulic network has very practical significance. In fact, it is necessary for supplying water to the users located along the pipe. In hydraulic model, demands are allocated to end nodes only for modelling purposes, even if users are located along pipes (see paper "Using EPANET for modelling water distribution systems with users along the pipes")."

→ We thank the reviewer for providing us with these interesting insights about demand modelling in hydraulic networks. In fact, our model could incorporate both – demands allocated at single nodes or demands located along the pipe. The latter model would translate to our framework through discretising the pipe such that it takes into account each individual consumer as a separate node. Then the transition to loop formation would translate as laying down the final segment that completes the pipe – thus closing the loop. To clarify the crucial difference between models allocating the demand at a single node and models taking into account demands located along the pipes, we added the Reference suggested by the referee to the modelling section of our revised manuscript along with the following statement:

"The in- and outflows may either represent individual supply nodes or allocated demands associated with the node [4]."

"In the field of water distribution network, branched networks hidden inside looped net-

works are searched for both modelling (e.g., papers "Comparison of newton-raphson global and loop algorithms for water distribution network resolution" and "Reformulated Co-Tree Flows Method Competitive with the Global Gradient Algorithm for Solving Water Distribution System Equations") and optimization (e.g., papers "The importance of the minimum path criterion in the design of water distribution networks" and "Fast network multi-objective design algorithm combined with an a posteriori procedure for reliability evaluation under various operational scenarios") purposes. Therefore, the tree network has only a theoretical significance in most cases."

→ We thank the reviewer for providing us with these interesting insights about the significance of tree networks in hydraulic engineering and for providing the corresponding references. In fact, we would like to point out that our main result is more general and may be extended beyond the first loop that forms: Our numerical results suggest that structural transitions are always discontinuous. To highlight these important results, we decided to split up Figure 3 into two separate Figures to illustrate the transition to loop formation also for links beyond the first loop. In the new Figure 4 and a new section, we now demonstrate that loops beyond the first one also form discontinuously, such that the discontinuous nature of the transition can be expected to hold for larger networks as well.

In the new section, we decided to add the interesting references suggested by the reviewer and discuss the theoretical importance of tree networks hidden in loopy networks as follows:

"Whereas the transition to the first loop that forms is important in many real-world supply networks, such as the *Gingko* leaf and the distribution grid shown in Figure 1, other networks display several loops, such that their formation beyond the first loop becomes important. In particular, the tree has mainly a theoretical importance in many applications such as hydraulic networks where spanning trees in loopy networks play an important role in modelling and optimisation [5–8]. Remarkably, we can demonstrate numerically that the discontinuous character of loop formation persists beyond the first loop."

"2 - I could see that the Authors set up an optimization problem with dissipation as objective function to minimize and budget constraints. In practical problems, such as water network design, budget is the objective function to minimize while there is a constraint on dissipation. Indeed, budget is the critical issue for engineers, which is then considered as objective function to minimize. How can this aspect be considered in your approach? Please

reply in the text."

→ We thank the reviewer for making us aware of this corresponding optimisation problem considered in practical settings. This interesting suggestion has led us to explore the corresponding optimisation problem for the five node network. In fact, we were able to demonstrate that the discontinous transition persists for problems where costs are minimised while the dissipation is constrained for both the edge-damage model and the fluctuating sink model. We included our results in a separate Supplementary Figure S5 and a Supplementary Note 5 and now comment on this problem in the main manuscript as follows:

"Note that different definitions of optimal networks arise in other applications, e.g. in hydraulic engineering where typically, the cost is minimised while the dissipation is constrained [7]. In a Supplementary Figure S5 and in Supplementary Note 5, we demonstrate that the same kind of discontinuous transition may be observed when extending our analysis to this setup."

Minor comments

"There are some oversights in the text"

"3 - lines 15-17. Indeed the trade-off is between cost and resilience."

→ We agree with this comment and changed it accordingly in the revised version of the manuscript. We also decided to refer to the trade-off between cost and resilience throughout the revised version of the manuscript and removed the reference to "efficiency" entirely since it was misleading.

"4 - line 21. "Establish""

→ We corrected this typo in the revised version of the manuscript.

"5 - Equation 2. Why is there "!"?"

→ In fact, the symbol "$\stackrel{!}{=}$" was meant to express that typically, the in- and outflows P are given and the flows are set to satisfy these in- and outflows. However, we agree that the notation was uncommon and the information already contained in the sentence before the Equation. We therefore decided to replace the symbol by a simple equality sign "=" in the revised version of the manuscript.

"6 - In text and equations, the Authors sometimes use "k_e" and other times "k". What is the difference?"

→ We thank the reviewer for pointing out this potential source of confusion to us. Unfortunately, it is unclear to us which usage of the symbol k the reviewer is referring to. In fact, we could not find any usage of the symbol k, but only the symbols κ - which represents the loopy capacity - and K which we use to denote the total available capacity. We have further clarified the usage of κ as the capacity of the first loop on page 3.

"7 - line 329 "updated""

→ We corrected this typo in the revised version of the manuscript.

REPLY TO REVIEWER 3

"I have reviewed the manuscript entitled "Discontinuous transition to loop formation in optimal supply networks," written by Franz Kaiser et al. This paper studies an interesting question: how to form loops in supply network? The authors discovered a discontinuous transition, which is very interesting. Furthermore, the authors derived the analytical solution. Yet, I do have several concerns and comments on the current form of the manuscript regarding the models, methods, and the results of this work. I am quite open to looking at a revised version if the author can address in a satisfactory fashion the issued discussed below:"

→ We thank the reviewer for the time taken to review our paper and for the opportunity to revise the manuscript. We are also grateful for the helpful comments which we address below point by point.

"In the abstract, it is unclear why adding new redundant edges is costly ye beneficial for resilience. How to define the resilience and cost of networks? There are many papers and also many definitions of these terms. I do not know what the definitions are here?"

→ We agree with the reviewer that our usage of the words "resilience" and "cost" was imprecise in the initial version of the manuscript. In the revised manuscript, we now state more clearly which definition of cost and resilience we assume here already in the introduction by adding the following statement:

"Here, we analyse the transition to loop formation on a theoretical basis and derive several analytical results. We consider optimal network structures in the sense that function is optimised while costs are constrained or vice versa. Two aspects of resilience are studied in detail – damage to edges and fluctuations of supply and demand. In particular, we investigate the optimal structure as a function of the severity of damage and the strength of fluctuations. "

Furthermore, we added more details about the cost parameter γ and its usage in practical applications when introducing the cost function to clarify what we mean by "cost". In addition to that, we added the following discussion of resilience after introducing the optimization problem in the modeling section:

”We focus on two different models here: a model with fluctuating sources and sinks and a model of stochastic damage to the edges. Both models may be considered to measure the network resilience: We call a network resilient if it is able to function properly under the uncertainities induced by edge damage or fluctuating inputs. For both models, our main question will be the following: Under which circumstances does the optimal network structure contain loops and how do these loops emerge?”

Finally, we substantially extended the introduction and the discussion to illustrate how our results relate to other concepts of resilience and cost. We hope that this clarifies the comment raised by the reviewer.

”More importantly, why is it important to study the effect of a single edge? As we know, the sizes of real-world networks are huge. The impact of a single link is almost nothing. Why should one care about the effect of a single edge on a huge network?”

→ We thank the reviewer for making us aware of the fact that single links may not be important when considering large networks. However, we would like to point out that our main result is more general and may be extended beyond the first loop that forms: Our numerical results suggest that structural transitions are always discontinuous. To highlight these important results, we decided to split up Figure 3 into two separate Figures to illustrate the transition to loop formation also for links beyond the first loop. In the new Figure 4 and a new section, we now demonstrate that in fact, loops beyond the first one also form discontinously, such that the discontinuous nature of the transition can be expected to hold for larger networks as well.

In addition to that, we would like to point out that single links may play a very important role when considering supply networks such as power grids. In a reanalysis of major blackouts, Pourbeik et al. [9] state the following about the role played by single transmission line failures in cascading outages: ”Typically, the blackout can be traced back to the outage of a single transmission (or generation) element.” This statement underlines the importance a single link may have – even in a larger network.

”The discontinuous bifurcation is very interesting. The authors studied the phase transition from no loop state to a single loop state. As we know, many real networks are sparse,

but they have loops. Why should one worry about this transition if one knows that the networks already have loops?"

→ We agree with the reviewer that it would be very interesting to extend our results to loop formation beyond the first loop. As discussed in the answer to the last question, we added a new Figure 4 and a new section to demonstrate that the transition to loop formation is always discontinous for the model class under consideration. Thus, the transition described in our paper remains relevant even if the network is already loopy.

In addition to that, the question *why and how* supply networks such as leaves form loops is an important question investigated in different recent publications. This is due to the fact that new, redundant connections in evolutionarily optimized networks are generally costly and thus their location must be chosen very carefully. In our manuscript, we suggest the stress relief mechanism – in addition to the increase in resilience – as yet another way to understand loop formation. To highlight this finding and embed it into a larger context, we extended the discussion significantly in that regard.

"In Eq. (2), it is unclear what is the meaning of $\stackrel{!}{=}$. *The authors give a reference to Eq. (3), but it is unclear why it is defined as this form. Eq. (4) is also confusing. The notations are hard to understand. For example, in Eq. (4), the authors use F(n,m) that corresponds to an edge between node m and n. But in Eq.(1) and (2), the authors used* $I_{n,e}$, *where n denotes a node, and e means an edge. The authors also define some terms with an index of link, for example,* k_e.*"*

→ We are sorry for this source of confusion. In the revised version of the manuscript, we avoid the symbol $\stackrel{!}{=}$ and use a simple equality sign "=" instead. We also revised the notation for the flows, now only using F_e and avoiding the notation $F_{(m,n)}$ entirely. We also completely revised the section where we present the model and separated it from the section discussing the optimisation problem.

"After Eq. (5), the authors described the F_e *and* $-F_e$. *It is still confusing how to choose the sign of* F_e. *Does it depend on the sign of* I *or on where are the source and sink?"*

→ Indeed, the orientation of the F_e is fixed by the incidence matrix I by virtue of Equation

(4). Here, since we are dealing with a single source, we adopt the convention that all edges are pointing away from the source as shown in Figure 2b, i.e. the flow is positive if flowing from the source to any other node. We agree that this was not stated clearly enough in the manuscript and thus revised it as follows:

"The resulting flows automatically satisfy Kirchhoff's voltage law (KVL) which states that the flow around any closed loop expressed in terms of the edges $C = \{e_{c_1}, e_{c_2}, ..., e_{c_{\max}}\}$ vanishes

$$\sum_{e \in C} z_e F_e = 0.$$

Here, the factor $z_e \in \{-1, 1\}$ is a prefactor used to keep track of the orientation of an edge e with respect to the orientation of the edge in the loop C, i.e.

$$z_e = \begin{cases} 1 & \text{if edge } e = (e_1, e_2) \text{ is oriented from } e_1 \text{ to } e_2, \\ -1 & \text{if edge } e = (e_1, e_2) \text{ is oriented from } e_2 \text{ to } e_1. \end{cases}$$

"

"The authors obtain Eq. (7) from Eq. (3). Is there any assumption that k_e is the same? Otherwise, how to get Eq. (7)? The equation after Eq. (11) seems reasonable to me. Why is the resource constraint cast into Eq. (8)?"

→ We are sorry for the unclear notation here. In fact, the average is taken over the flows only since the fluctuations on the sinks are assumed to affect the flows by virtue of Eq.(2) and thus the nodal potentials by virtue of Eq.(3), whereas the network topology is assumed to be fixed by the construction of the network. We then optimise the network structure given the fluctuations. This is in contrast to the edge-damage model, where the average also takes into account explicitly the capacities, since they are directly modified in this case. To clarify this important fact, we added a statement to the revised version of the manuscript after the new Eq.(8).

The resource constraint as presented in Eq.(8) (Eq.(6) in the revised version of the manuscript) allows to describe different situations that occur frequently in real-world supply networks. To clarify this functional form in the revised version of the manuscript, we added a detailed discussion of the cost parameter γ for different possible applications.

"The statement in the first two sentences on page 3 is not true. Most of the real-world networks do not have geometric constrains. Is this tool only useful for the spatially embedded networks without any long-range links?"

→ While most supply networks are spatially embedded and thus geometrically constrained, indeed, this is not generally true for arbitrary networks as pointed out by the reviewer. Our approach is not fundamentally limited to planar networks without long-range links. We illustrate a possible application to the simplest non-planar network, K_5, the complete graph on five nodes, in a separate Supplemental Figure S6, which naturally extends the five node network analysed throughout the manuscript. Nevertheless, we would like to point out that the theoretical analysis - in contrast to the numerical analysis - is greatly simplified for planar networks due to the fact that the cycle flows can be uniquely determined, whereas non-planar networks require a non-unique embedding of the network onto surfaces with a non-zero genus to determine the cycle basis (see e.g. Ref. [10]). To highlight that the analysis may also be applied to non-planar networks, we added the following statement main text after introducing the geometric constraints:

"Note that while planarity of the network described by the set of potential edges $\mathcal{E}$ simplifies the theoretical analysis, our results are not limited to planar networks as we demonstrate for a simple, non-planar network in Supplementary Figure S6."

---

[1] F. Corson, Fluctuations and redundancy in optimal transport networks, Physical Review Letters **104**, 048703 (2010).

[2] E. Katifori, G. J. Szöllősi, and M. O. Magnasco, Damage and fluctuations induce loops in optimal transport networks, Physical Review Letters **104**, 048704 (2010).

[3] S. Bohn and M. O. Magnasco, Structure, Scaling, and Phase Transition in the Optimal Transport Network, Physical Review Letters **98**, 088702 (2007).

[4] G. Farina, E. Creaco, and M. Franchini, Using EPANET for modelling water distribution systems with users along the pipes, Civil Engineering and Environmental Systems **31**, 36 (2014).

[5] S. Elhay, A. R. Simpson, J. Deuerlein, B. Alexander, and W. H. A. Schilders, Reformulated

Co-Tree Flows Method Competitive with the Global Gradient Algorithm for Solving Water Distribution System Equations, Journal of Water Resources Planning and Management **140**, 04014040 (2014).

[6] E. Creaco and M. Franchini, Comparison of Newton-Raphson Global and Loop Algorithms for Water Distribution Network Resolution, Journal of Hydraulic Engineering **140**, 313 (2014).

[7] E. Creaco and M. Franchini, Fast network multi-objective design algorithm combined with an a posteriori procedure for reliability evaluation under various operational scenarios, Urban Water Journal **9**, 385 (2012).

[8] C. Ciaponi, E. Creaco, L. Franchioli, and S. Papiri, The importance of the minimum path criterion in the design of water distribution networks, Water Supply **17**, 1558 (2017).

[9] P. Pourbeik, P. Kundur, and C. Taylor, The anatomy of a power grid blackout - root causes and dynamics of recent major blackouts, IEEE Power and Energy Magazine **4**, 22 (2006).

[10] H. Ronellenfitsch, D. Manik, J. Hörsch, T. Brown, and D. Witthaut, Dual Theory of Transmission Line Outages, IEEE Transactions on Power Systems **32**, 4060 (2017).

Reviewer #1 (Remarks to the Author):

I would like to thank the authors for their efforts in revising the manuscript. The updated version has been greatly improved and my concerns have been adequately addressed.

Reviewer #2 (Remarks to the Author):

The Authors have replied successfully to all my comments. The paper can be accepted.

Reviewer #3 (Remarks to the Author):

The authors have addressed all of my previous comments. The author added Fig. 4 to use a small network to illustrate the case of multiple loops. Here, I only have a few additional comments regarding the new figure.

The authors only show the capacity as a function cost. To better compare with the first loop results, it is reasonable to show the fluctuation parameter and damage parameter's effects, as in Fig. 3.

Were the critical points obtained in Fig. 4 numerically? It seems that the curves are very different compared Fig. 3.

Response to Reviewers for:

Discontinuous transition to loop formation in optimal supply networks

Franz Kaiser,[1,2,*] Henrik Ronellenfitsch,[3,4] and Dirk Witthaut[1,2,†]

[1]*Forschungszentrum Jülich, Institute for Energy and Climate Research (IEK-STE), 52428 Jülich, Germany*

[2]*Institute for Theoretical Physics, University of Cologne, Köln, 50937, Germany*

[3]*Department of Mathematics, Massachusetts Institute of Technology, Cambridge, MA 02139, U.S.A.*

[4]*Physics Department, Williams College, 33 Lab Campus Drive, Williamstown, MA 01267, U.S.A.*

* f.kaiser@fz-juelich.de

† d.witthaut@fz-juelich.de

REPLY TO REVIEWER 1

" I would like to thank the authors for their efforts in revising the manuscript. The updated version has been greatly improved and my concerns have been adequately addressed."

→ We thank the reviewer for this positive assessment and are glad that she/he likes the changes we have made.

REPLY TO REVIEWER 2

"The Authors have replied successfully to all my comments. The paper can be accepted."

→ We thank the reviewer for this positive assessment and are glad that she/he likes the changes we have made.

REPLY TO REVIEWER 3

"The authors have addressed all of my previous comments. The author added Fig. 4 to use a small network to illustrate the case of multiple loops. Here, I only have a few additional comments regarding the new figure."

→ We thank the reviewer for the positive assessment and are happy that she/he likes the changes we have done. We address the final comments below.

"Were the critical points obtained in Fig. 4 numerically? It seems that the curves are very different compared Fig. 3."

→ In fact, the critical points in both Figure 3 and Figure 4 were obtained numerically. The main difference is the fact that we solved the set of equations resulting from the KKT conditions to find all potential minima to produce Figure 3, but this is no longer possible for the network shown in Figure 4. Furthermore, the reason why the critical cost parameters differ in both cases is the difference in the network topologies considered in both figures. In Figure 3, we analyse the much smaller network shown in Figure 2a, consisting of only five nodes and four to five edges. The network analysed in Figure 4 has 36 edges and 37 nodes and has a set of 90 potential edges. Since the overall available capacity is constrained to

$K = 1$ by the resource constraint

$$\sum_{e\in E} k_e^\gamma = K^\gamma$$

in both cases, the capacity available for a single link differs largely between the two networks such that the curves are very different, but the discontinuous transition persists.

"The authors only show the capacity as a function cost. To better compare with the first loop results, it is reasonable to show the fluctuation parameter and damage parameter's effects, as in Fig. 3."

→ We agree that it would be nice to compare the results. However, we decided to leave the Figure as it is since we introduced it to increase the accessibility of our results and we are afraid that introducing additional complexity would make the Figure more complicated. Nevertheless, we decided to add an additional Supplementary Figure 8 where we demonstrate the analogous transition beyond the first loop for varying fluctuation strengths and fixed cost.

We decided not to discuss the damage model here for the following reason: Whereas we obtained the locally optimal capacities in Figure 3 by explicitly solving the set of Equations that results from the KKT conditions, this is no longer feasible for the setup in Figure 4. We therefore used an iterative approach developed in Ref. [1] as described in the Methods section. To clarify this, we added the following statement to the section discussing Figure 4:

"Note that in contrast to Figure 3, the optimal capacities are obtained here using an iterative approach for finding local minima of the dissipation that is due to Ref. [1] (see Methods). In a Supplementary Figure 8, we demonstrate that an analogous transition exists for varying fluctuation strength σ and fixed cost parameter γ."

Finally, as outlined in the last comment, we would like to point out that the networks under examination are vastly different such that the results are not directly comparable.

---

[1] Corson, F. Fluctuations and redundancy in optimal transport networks. *Phys. Rev. Lett.* **104**, 048703 (2010).
